# Enhancement of Outflow Facility in the Murine Eye by Targeting Selected Tight-Junctions of Schlemm’s Canal Endothelia

**DOI:** 10.1038/srep40717

**Published:** 2017-01-16

**Authors:** Lawrence C. S. Tam, Ester Reina-Torres, Joseph M. Sherwood, Paul S. Cassidy, Darragh E. Crosbie, Elke Lütjen-Drecoll, Cassandra Flügel-Koch, Kristin Perkumas, Marian M. Humphries, Anna-Sophia Kiang, Jeffrey O’Callaghan, John J. Callanan, A. Thomas Read, C. Ross Ethier, Colm O’Brien, Matthew Lawrence, Matthew Campbell, W. Daniel Stamer, Darryl R. Overby, Pete Humphries

**Affiliations:** 1 Neurovascular Genetics, Smurfit Institute of Genetics, Trinity College, University of Dublin, Dublin 2, Ireland; 2 Department of Bioengineering, Imperial College London, London, UK; 3 Department of Anatomy, University of Erlangen-Nürnberg, Erlangen, Germany; 4 Department of Ophthalmology, Duke University, Durham, NC, USA; 5 Ross University School of Veterinary Medicine, P. O. Box 334, Basseterre, St. Kitts, West Indies; 6 Department of Ophthalmology and Vision Sciences, University of Toronto, Canada; 7 Coulter Department of Biomedical Engineering, Georgia Institute of Technology and Emory University, Atlanta, USA; 8 Ophthalmology, Mater Hospital, UCD School of Medicine, Dublin, Ireland; 9 RxGen, Hamden, CT, USA

## Abstract

The juxtacanalicular connective tissue of the trabecular meshwork together with inner wall endothelium of Schlemm’s canal (SC) provide the bulk of resistance to aqueous outflow from the anterior chamber. Endothelial cells lining SC elaborate tight junctions (TJs), down-regulation of which may widen paracellular spaces between cells, allowing greater fluid outflow. We observed significant increase in paracellular permeability following siRNA-mediated suppression of TJ transcripts, claudin-11, zonula-occludens-1 (ZO-1) and tricellulin in human SC endothelial monolayers. In mice claudin-11 was not detected, but intracameral injection of siRNAs targeting ZO-1 and tricellulin increased outflow facility significantly. Structural qualitative and quantitative analysis of SC inner wall by transmission electron microscopy revealed significantly more open clefts between endothelial cells treated with targeting, as opposed to non-targeting siRNA. These data substantiate the concept that the continuity of SC endothelium is an important determinant of outflow resistance, and suggest that SC endothelial TJs represent a specific target for enhancement of aqueous movement through the conventional outflow system.

Under physiological conditions, the majority of aqueous humour (AH) exits the anterior chamber through the conventional outflow pathway in humans^1^,^2^,^3^. In this pathway, AH filters sequentially through the trabecular meshwork (TM), including the juxtacanalicular tissue (JCT), and the endothelial lining of Schlemm’s canal (SC) before entering the SC lumen and draining into the episcleral veins. Electron microscopic evidence has indicated that AH drainage across SC endothelium occurs through micron-sized pores that pass either through (transcellular) or between (paracellular) individual SC cells^4^,^5^,^6^,^7^,^8^,^9^. In particular, a significant fraction of AH crosses the inner wall of SC via paracellular pores^10^. Moreover, the presence of tight-, adherens- and gap-junctions in SC endothelial cells provides a mechanism by which the conventional outflow pathway is dynamically responsive to constantly changing physiological conditions while still preserving the blood-aqueous barrier^11^,^12^,^13^,^14^,^15^,^16^,^17^. It has long been recognised that elevated intraocular pressure (IOP) associated with primary open-angle glaucoma (POAG) is due to elevated resistance to AH outflow through the conventional outflow pathway^18^, although the cause of elevated outflow resistance in glaucoma remains to be fully elucidated. Previous studies support the concept that outflow resistance is modulated through a synergistic hydrodynamic interaction between JCT and SC endothelium such that inner wall pore density may influence outflow resistance generation by defining the regions of filtration through the JCT^19^,^20^,^21^. As glaucomatous eyes have reduced SC inner wall pore density, decreased porosity of the inner wall appears to contribute to elevated outflow resistance and increased IOP^22^,^23^,^24^.

Prolonged elevation of IOP results in progressive degeneration of retinal ganglion cell axons, and hence to irreversible vision loss. Treatment of POAG by lowering IOP remains the only approach to limiting disease progression. Topically applied medications that either reduce AH production or increase drainage through the unconventional (uveoscleral) outflow pathway are widely used in management of IOP in patients with POAG^25^. However, a proportion of patients do not respond optimally to such medications and, therefore, there is a clear need to investigate novel approaches to reduce outflow resistance by identifying specific targets within the conventional outflow pathway through which this might be achieved. Owing to the fact that a major fraction of AH filtration at the level of SC appears to largely pass through paracellular routes^10^, strategies specifically targeting cell-cell junctions between endothelial cells of the inner wall of SC may be effective at decreasing outflow resistance. Hence, we hypothesised that down-regulation of selected tight junction (TJ) components of endothelial cells lining the inner wall of SC may increase the paracellular spaces between these cells, facilitating flow of AH across the inner wall into the SC (Fig. 1), thus reducing outflow resistance and IOP.

In this report, we have identified TJ components in human primary cultures of SC endothelial cells (SCEC), and also in mouse and non-human primate outflow tissues. We show that siRNA-mediated down-regulation of such components increases the paracellular permeability of human primary SCEC monolayers to 70 kDa FITC-dextran, and decreases transendothelial electrical resistance. Furthermore, intracameral delivery of siRNAs targeting selected TJ components is shown to increase intercellular open spaces between SC inner wall endothelial cells as observed by transmission electron microscopy (TEM) and elevates outflow facility (the mathematical inverse of outflow resistance) in normotensive mice. In summary, our findings clearly identify a specific approach to promoting AH outflow by direct manipulation of selected TJs within the conventional outflow pathway.

## Results

### Characterisation of tight junction expression in human SC endothelial cells

**Figure 2:**
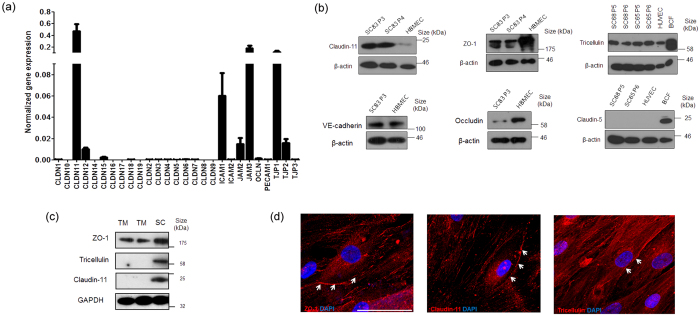
Characterisation of tight junction expression in human Schlemm’s canal endothelial cells. **(a)** The human TJs RT^2^ Profiler PCR array was used to profile the expression of claudin and adhesion junctional proteins. Bar graphs illustrate average relative gene expression (2^−ΔCT^) normalised to 5 housekeeping genes from 4 different human SCEC strains. Data are mean ± s.e.m. Note the break in scale for normalised gene expression. **(b)** Protein analysis of claudin-11, ZO-1, tricellulin, VE-cadherin, occludin and claudin-5 in cultured human SCEC. HBMEC = human brain microvascular endothelial cells; BCF = Mouse brain capillary fraction; B-actin as loading control. Different SCEC strains are denoted followed by passage (P) number. **(c)** Tight junction protein expression in TM (TM120 and TM130) and SCEC. GAPDH as loading control. **(d)** White arrow heads illustrate immuno-detection of ZO-1, claudin-11 and tricellulin (Cy3) in cultured human SCEC. Blue = DAPI nuclei staining. Scale bar, 50 μm.

We examined the TJ expression profile in primary cultures of human SCEC isolated from four individual donors, with the objective of determining key junctional components that regulate permeability and selectivity of the inner wall of SC. The mean normalised expression (2^−∆∆Ct^) of genes encoding claudin and adhesion junctional proteins from four different SCEC strains is shown in Fig. 2a. The complete expression pattern can be found as Supplementary Fig. S1. The expression profile shows that claudin-11 (or oligodendrocyte specific protein) was amongst the highest expressed claudin-based TJ protein in cultured SCEC (Fig. 2a). In addition, zonula-occludens-1 protein (ZO-1, also known as *TJP1*), a key component of junctional complexes that regulate TJ formation, was also expressed at high levels in cultured SCEC. The cell-cell adhesion molecule, junctional adhesion molecule-3 (JAM3) was also highly expressed in human SCEC monolayers. In contrast, occludin and claudin-5, which are major TJ components of human and mouse brain and inner retinal vascular endothelium^26^,^27^ were expressed at low levels in human SCEC. Collectively, these data indicate that claudin-11 is the dominant claudin in the TJs of cultured SCEC, and that ZO-1 is a major junctional associated protein of cultured SCEC. We also compared transcript levels of claudin-11 and ZO-1 in cultured monolayers of human SCEC (SC77) against those of human TM cells (TM93), and observed expression levels of claudin-11 to be 2.52-fold higher in SCEC than in TM cells (Supplementary Fig. S2). However, no significant difference in ZO-1 transcript expression was observed between TM and SCEC.

Claudin-11 and ZO-1 protein expression was detected in cultured SCEC by Western blot (Fig. 2b). In addition, we also detected expression of another TJ protein, tricellulin (also known as MARVELD2) in cultured SCEC, which was not included in the PCR array. Consistent with previous studies^14^,^28^, expression of vascular endothelial (VE)-cadherin was also identified in cultured SCEC (Fig. 2b). However, we did not detect claudin-5 protein expression in cultured SCEC, and only low levels of occludin protein expression were detected, an observation consistent with the PCR array data. Furthermore, we did not detect claudin-11 and tricellulin expression in TM cells (TM120 and 130), whereas both TM and SCEC (SC82) were shown to express ZO-1 protein (Fig. 2c). This is consistent with a previous finding showing that both TM and SCEC express the junction-associated protein, ZO-1^29^. Immunocytochemistry was then undertaken to examine the expression patterns of TJ proteins in confluent SCEC monolayers. We observed discontinuous membrane-specific staining patterns for ZO-1, claudin-11 and tricellulin in cultured SCEC monolayers (Fig. 2d).

### Characterisation of expression of tight junction and tight junction associated components in mouse and non-human primate outflow tissues

**Figure 3:**
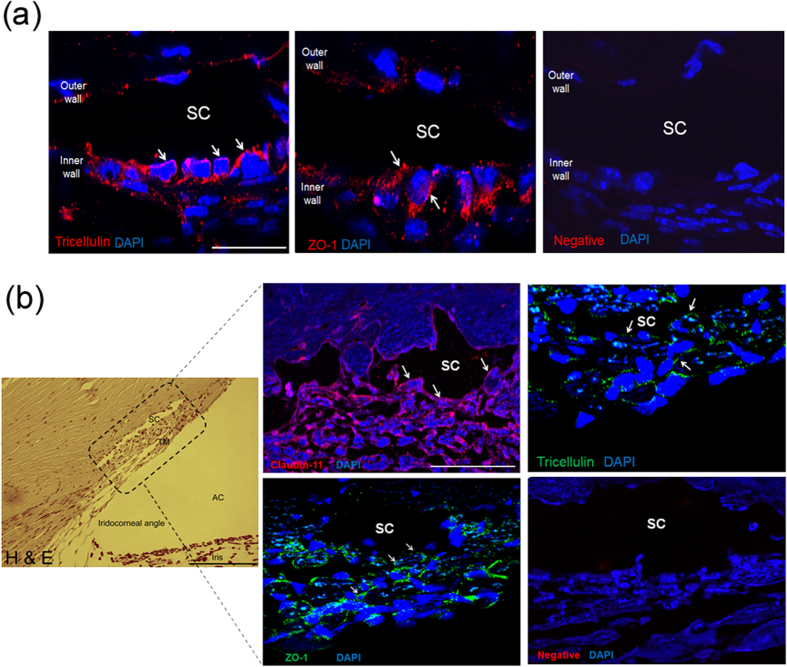
Characterisation of tight junction expression in mouse and non-human primate outflow tissues. (**a)** Immunostaining of tricellulin and ZO-1 in frozen sections of mouse anterior segments. ZO-1 and tricellulin = Cy3 (red); DAPI = blue; SC = Schlemm’s canal lumen. Scale bar, 50 μm. (**b)** H&E staining of paraffin monkey anterior segments (left panel). Boxed area depicts superimposed regions shown in immunofluorescence images. AC = anterior chamber; SC = Schlemm’s canal lumen; TM = trabecular meshwork. Scale bar, 200 μm. Immunofluorescent images of claudin-11, ZO-1 and tricellulin staining in the inner wall endothelium of SC. White arrows indicate detection of corresponding tight junctions at the inner wall of SC endothelium. Negative = no primary antibody. Scale bar, 50 μm.

We performed immunohistochemistry (IHC) on frozen sections of mouse anterior segments to localise the expression of TJ proteins in the outflow region comprising the TM and the inner wall of SC. Immunofluorescent images show tricellulin and ZO-1 staining predominantly localising in the inner wall endothelium of SC (Fig. 3a). In particular, we observed ZO-1 staining to be diffusely distributed in the cytoplasm of SCEC. In regions where part of the endothelium was cut obliquely to the inner wall of SC, continuous junctional strands were displayed around SCEC margins. ZO-1 and tricellulin staining were also detected in the TM region and in the outer wall. In both regions the endothelial cells were connected by TJs. However, we did not detect claudin-11 or claudin-5 staining in the inner wall of SC and TM with the antibodies used in this study (Supplementary Fig. S3). These data indicate that murine outflow tissues may possess a different junctional composition at the inner wall of SC as compared to humans, with the possible absence of claudin-based tight junctional proteins in TM and SCEC. However, the presence of ZO-1 and tricellulin along the inner wall in mice indicates that these proteins may be suitable targets for assessment of effects of TJ down-regulation in mice.

IHC was performed on paraffin sections of African green monkey anterior segments to identify the junctional composition of the outflow region. Hematoxylin and eosin staining (H&E) of the anterior chamber clearly identified the iridocorneal angle and conventional outflow tissues (Fig. 3b). Superimposed immunofluorescent imaging showed strong continuous claudin-11 staining along the endothelial cell margins of the inner wall of SC, highly indicative of TJ barrier function (Fig. 3b). Claudin-11 immunostaining was also present along the outer wall of SC and between TM cells. Similarly, ZO-1 and tricellulin staining were observed in the inner wall endothelium of SC. All three TJ proteins were present between TM endothelial cells, but the staining was less intense than in the inner wall endothelium. In addition, we did not detect claudin-5 expression in SCEC isolated from non-human primates (Supplementary Fig. 4). These data indicate that SCEC in non-human primates possess a similar TJ barrier composition to that found in humans.

### Validation of tight junction siRNAs

**Figure 4:**
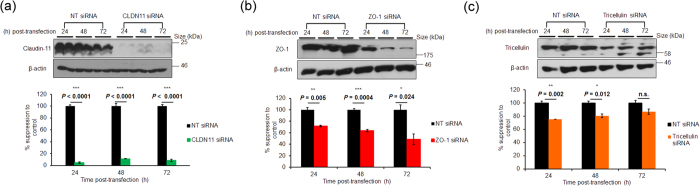
siRNA-mediated down-regulation of tight junction RNA transcripts in cultured human SCEC. Representative Western blots of (**a)** claudin-11, (**b)** ZO-1 and (**c)** tricellulin knockdown in cultured human SCEC over a 72 h period. Corresponding bar graphs depict densitometric analysis of percentage protein normalised to β-actin. NT siRNA = non-targeting siRNA. Data are mean ± s.e.m.; n.s. = *P* ≥ 0.05 (n = 4, unpaired *t*-test).

In order to validate the suppression efficiency of pre-designed siRNAs targeting the human transcripts of claudin-11, ZO-1 and tricellulin, cultured SCEC were separately transfected with 40 nM of each siRNA, and levels of endogenous TJ expression were assessed in a time-dependent manner by Western blot. Time-dependent down-regulation of claudin-11 expression to 5 ± 3% (p < 0.0001), 11 ± 1% (p < 0.0001) and 9 ± 4% (p < 0.0001) (mean ± s.e.m.), was achieved at 24, 48 and 72 h post-transfection respectively, as compared to non-targeting (NT) siRNA (Fig. 4a). ZO-1 expression was reduced to 72 ± 3% (p = 0.005), 64 ± 4% (p = 0.0004) and 49 ± 18% (p = 0.02) at 24, 48 and 72 h post-transfection respectively (Fig. 4b). Furthermore, tricellulin expression was reduced to 75 ± 0.2% (p = 0.002), 81 ± 6% (p = 0.012) and 87 ± 8% (p > 0.05) at 24, 48 and 72 h respectively following siRNA treatment (Fig. 4c). The difference in knockdown efficiencies likely indicates that ZO-1 and tricellulin have slower protein turnover rates than claudin-11 in cultured SCEC. A cell viability assay was performed on transfected SCEC and no change in viability due to siRNA treatment was detected when cells were treated with either 40 or 200 nM of siRNA (Supplementary Fig. S5). siRNAs targeting mouse ZO-1 and tricellulin were also validated and show efficient knockdown of gene expression *in vitro* (Supplementary Fig. S6).

The efficacy of siRNA inhibition *in vivo* was tested in retinas from mice injected intravitreally with siRNA against ZO-1 and tricellulin. RT-PCR carried out on RNA extracted from mouse retinas showed that 12 hr post-injection, tricellulin RNA was significantly reduced to 0.32 fold (p = 0.049; Supplementary Fig. S7) compared to eyes injected with NT-siRNA while ZO-1 was reduced to 0.57 fold (p = 0.048; Supplementary Fig. S7). This approach using retina was taken because of the difficulty in isolating SC endothelium from mouse eyes to perform a reliable quantification analysis.

We performed cell death assays to assess SCEC viability following siRNA inoculation *in vivo* in mouse outflow tissues. Immunohistochemistry was performed on eyes 48 hours post injection with either targeting or NT siRNA. Approximately 30–40 12 μm sections were each stained by TUNEL and complemented by cleaved caspase-3 staining as markers of apoptosis for representatives of targeting and non-targeting siRNA. For TUNEL staining, most sections displayed some apoptotic damage in the corneal epithelium. Parts of the ciliary body were also the site of minor labelling, regardless of treatment received. Closer inspection of the angle and outflow tissue itself provided no evidence of any apoptotic cell death in either targeting or NT controls. Cleaved caspase-3 was sparsely detected in the ciliary body, and effectively absent in the angle or outflow tissue in either treatment, correlating with observations by TUNEL (Supplementary Fig. S8).

### Effect of down-regulation of tight junctions on SCEC monolayer permeability

**Figure 5:**
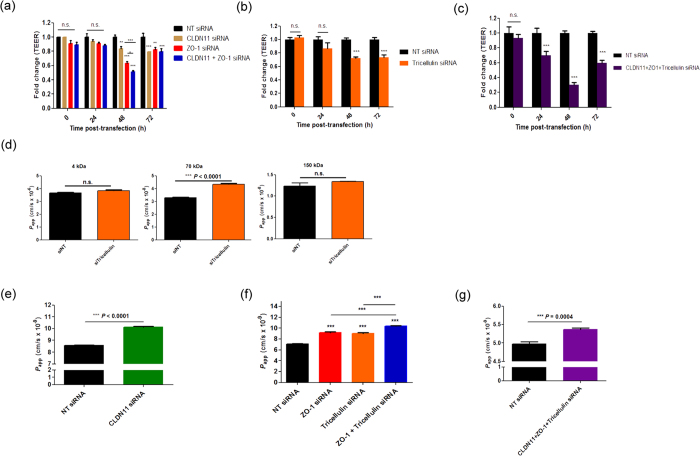
siRNA-mediated down-regulation of tight junction RNA transcripts modulates TEER and paracellular permeability in cultured SCEC monolayers. (**a**) Effect of siRNA-mediated knockdown of TJ RNA transcripts on TEER across human SCEC monolayers. 40 nM of siRNA targeting claudin-11, ZO-1, or in combination were transfected into human SCEC, and TEER was measured 24, 48 and 72 h post-transfection. *P < 0.05, **P < 0.01, ***P < 0.001, n.s. *P* ≥ 0.05 (n = 3 separate cell transfection, two way analysis of variance (ANOVA) followed by Bonferroni’s multiple comparison post-tests). Data are fold change ± s.e.m. (**b**) TEER measurements following treatment with tricellulin siRNAs in cultured SCEC monolayers (n = 5 separate cell transfections, two way ANOVA followed by Bonferroni’s multiple comparison post-tests). ***P < 0.0001. Data are fold change ± s.e.m. (**c**) TEER measurements following treatment of SCEC monolayers with a combination of claudin-11, ZO-1 and tricellulin siRNAs (n = 7 separate cell transfections, two way ANOVA followed by Bonferroni’s multiple comparison post-tests). ***P < 0.001. n.s. = *P* > 0.05. (**d**) Cultured SCEC monolayers demonstrate size selectivity. Apparent permeability co-efficient (*P*_*app*_, cm/s) of 4, 70 and 150 kDa FITC dextrans was determined following treatment with siRNAs targeting tricellulin. (***p < 0.0001; n = 3). n.s. = *P* ≥ 0.05 (**e**,**f**,**g**) *P*_*app*_ of 70 kDa FITC-dextran through human SCEC monolayers following treatment with claudin-11, ZO-1 and tricellulin siRNAs, or in combination. NT = non-targeting. Data are mean ± s.e.m. Note the break in scale for *P*_*app*_ (**e**,**f**). (**e**) ***P < 0.0001, n = 6. (**f**) (***P < 0.0001, n = 4). (**g**) ***P = 0.0004, n = 6. (unpaired Student’s *t*-test for left and right bar graphs; one way ANOVA followed by Bonferroni’s *post hoc* test for middle bar graphs).

In order to address the hypothesis that down-regulation of TJ components in SCEC could be used as a means of modulating the resistance of SC inner wall, transendothelial electrical resistance (TEER) was measured to assess changes in endothelial barrier function in confluent SCEC monolayers following TJ knockdown. SCEC monolayers transfected with claudin-11 or ZO-1 siRNAs showed significant reduction in TEER compared to NT siRNAs at 48 and 72 h post-transfection (p < 0.001; Fig. 5a). Furthermore, transfection with a combination of claudin-11 and ZO-1 siRNAs elicited a significant decrease in TEER, and the magnitude of decrease was more profound than those treated with single siRNAs at 48 h post-transfection (p < 0.001, Fig. 5a). Similarly, treatment with tricellulin siRNA alone also showed significant reduction of TEER at 48 h post-transfection, and the effect was sustained up to 72 h (p < 0.001, Fig. 5b). We next treated SCEC monolayer simultaneously with a combination of three siRNAs targeting claudin-11, ZO-1 and tricellulin, and observed significant reduction in TEER from 24 to 72 h post-transfection as compared to control (p < 0.001, Fig. 5c). Measured TEER values can be seen in Supplementary Table S1.

The effect of TJ down-regulation on endothelial permeability was tested in confluent SCEC monolayers using non-ionic macromolecular tracer, FITC-dextran (FD), which can only transverse via the paracellular route. To investigate the size selectivity of paracellular permeability in SCEC monolayers, we first determined the flux of 4, 70 and 150 kDa FD in the basal to apical direction following treatment of monolayers with siRNAs targeting tricellulin. At 24 h post-transfection, we observed no difference between control and treated in apparent permeability co-efficient (*P*_*app*_) to the 4 kDa FD (≈3.66 × 10^−6^ cm/s), which readily passes through the monolayer. In contrast, the 150 kDa FD did not readily cross the monolayer (≈1.23 × 10^−8^ cm/s). The largest decrease in barrier tightness as measured by *P*_*app*_ was observed with the 70 kDa FD (p < 0.0001, Fig. 5d). These data indicate that down-regulation of tricellulin in SCEC monolayers selectively opens the paracellular route to macromolecules of 70 kDa. Following this, we treated SCEC monolayers with siRNAs targeting other TJs, and observed that down-regulation of claudin-11 (p < 0.0001), ZO-1 (p < 0.0001), as well as tricellulin (p < 0.0001) significantly increased paracellular flux of 70 kDa FD, as compared to controls (Fig. 5e,f). In addition, *P*_*app*_ (70 kDa FD) was observed to be significantly greater in monolayers treated with a combination of ZO-1 and tricellulin siRNAs than control (p < 0.0001), and compared to those treated singly with either ZO-1 or tricellulin siRNA (p < 0.001) (Fig. 5f). Furthermore, treatment with a combination of siRNAs targeting three TJs simultaneously also increased *P*_app_ of SCEC to 70 kDa FD (p = 0.0004 vs. control; Fig. 5g). We used primary SCEC strains from different donor eyes for flux assays in Fig. 5e–g, and as a consequence, we observed natural variability in baseline *P*_*app*_ values and responses to TJ down-regulation from different strains. Collectively, these data demonstrate that claudin-11, ZO-1 and tricellulin contribute to the barrier function of cultured human SCEC, and that siRNA-mediated down-regulation of these cellular junctional proteins significantly alters endothelial cell barrier integrity and permeability.

### Ultrastructural analysis of the inner wall endothelium of SC following treatment with siRNAs

**Figure 6:**
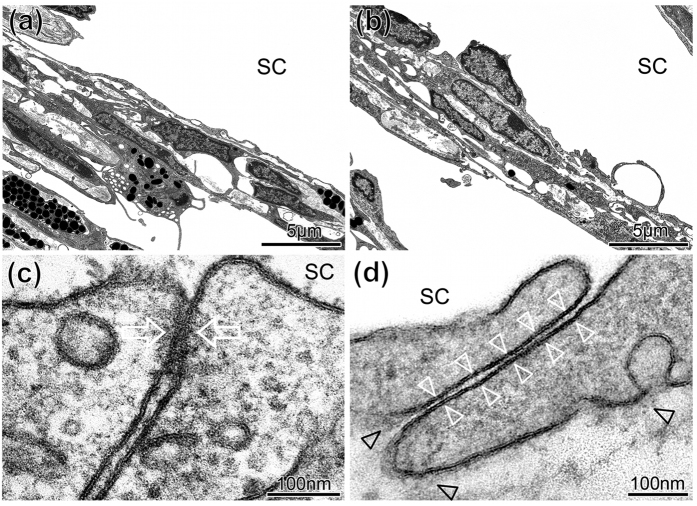
Transmission electron microscopic analysis of sagittal sections of the inner wall of SC following siRNA treatment. (**a,b)** Representative sagittal sections through the inner wall of Schlemm’s canal (SC) and outer trabecular meshwork (TM) of a mouse eye treated with **(a)** non-targeting (NT) or **(b)** targeting (T) siRNA illustrating intact cells and an intact and continuous inner wall endothelium that appeared similar in both cases. The inner wall endothelium is connected to the underlying ECM so that no ballooning was visible. (**c**,**d**) High magnifications of sagittal sections through intercellular clefts along the inner wall endothelium of SC showing examples for junctions quantitatively evaluated as closed **(c)** with fusion between the neighbouring cell membranes (arrows) or open clefts **(d)** where the cell membranes of adjacent endothelial cells were clearly separated along the entire cleft length (white arrowheads). Despite the open clefts, adhesions to subendothelial matrix (black arrowheads) were preserved. The number of open intercellular clefts was quantified (see Tables 1 and 2).

To examine how siRNA treatment affects the continuity of the inner wall of SC, ultrastructural investigation of TJs between SC cells was performed by TEM. Six wild type C57BL/6J mice were intracamerally injected with a combination of 1 μg ZO-1 siRNA and 1 μg of tricellulin siRNA, and contralateral eyes were injected with 2 μg of NT siRNA. 48 h post-injection, all animals were sacrificed with eyes enucleated immediately after death and immersed for TEM investigation. As can be seen in Fig. 6a,b, the inner wall of SC in both treated and control eyes appeared similar. The inner wall was continuous without loss of cells or apparent cellular damage, and in both control and treated eyes there were no swollen cells that would indicate necrosis. There were also no cellular extensions and nuclear densifications or fragmentations that would indicate apoptosis.

To more clearly visualize cell membranes and junctions, sections were stained with UAR-EMS rather than uranyl acetate (see materials and methods). This staining allowed better visualization of the intercellular junctions and revealed that intercellular clefts between neighbouring SC cells were more often open in eyes treated with targeting siRNAs than in controls, indicating an absence or weakening of the TJ complexes. Open clefts exhibited a typical width of 10–20 nm without any contact between neighbouring cell membranes, while closed clefts exhibited a focal fusion between neighbouring cell membranes often surrounded by small cytoplasmic filaments (Fig. 6c,d). Quantification of intercellular junctions was performed by 2 independent observers, who examined TEM sections at 80,000x along the anterior-posterior extent of the inner wall from 4 regions of each eye (n = 6 treated and 5 control eyes; one control eye was removed as, for technical reasons, not all 4 regions could be evaluated). Each section contained between 10–30 cells, and each region was separated from another by at least several hundred microns, such that each region could be considered an independent sample. This quantification revealed that approximately 33% of intercellular junctions were open in eyes treated with targeting siRNA (Table 1). In contrast, only approximately 2% of intercellular junctions were open in contralateral eyes treated with non-targeting siRNA (Table 2), and this difference was statistically significant (p = 0.004, unpaired Student’s t-test). These data reveal that the siRNA treatment is opening intercellular clefts along the inner wall of SC *in vivo*, presumably by affecting TJs.

It is feasible that siRNA treatment may also have affected adherens or cell-matrix junctions that provide mechanical support between endothelial cells and to the subendothelial tissue. Indeed, TJs and adherens junctions are coupled, and disassembly of adherens junctions often leads to disassembly of TJ^30^. To determine whether siRNA had affected these other junctional types, we examined for disconnections between the inner wall and subendothelial tissue that typically leads to inner wall ‘ballooning’ as observed following treatment with Na_2_-EDTA^31^,^32^. In none of the 4 regions examined per eye in either case, did we observe any ballooning of the inner wall (Fig. 6a,b). Even in areas with open intercellular clefts, the basal cell membranes of the endothelial cells were still attached to the underlying extracellular matrix (ECM) (Fig. 6d). This implies that the cell-matrix adhesions remained intact. We also examined for adhered platelets, which is a sign of inner wall damage, as platelets often seal endothelial gaps where ECM is exposed to the lumen of SC^31^,^32^. No adhering platelets were observed in any region of any eye.

### Effect of down-regulation of tight junctions on outflow facility *ex vivo*

**Figure 7:**
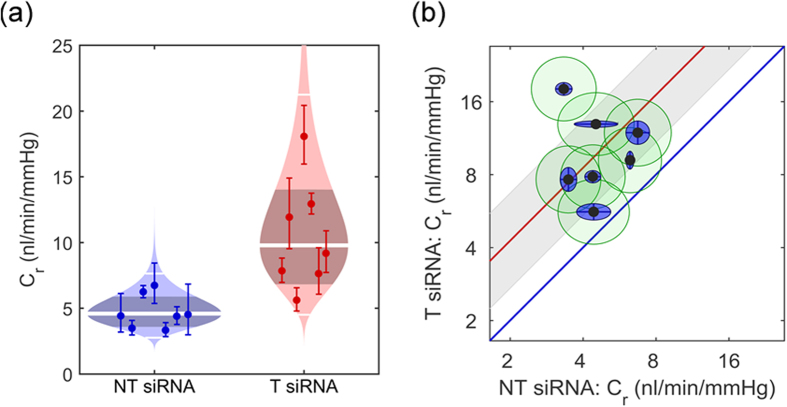
Effect of down-regulation of tight junction RNA transcripts on outflow facility *ex vivo*. **(a)** ‘Cello’ plots showing the individual values and statistical distribution of outflow facility at 8 mmHg (*C*_*r*_) for eyes treated with either non-targeting (NT) siRNA or a combination of ZO-1 and tricellulin targeting (T) siRNA. Each individual point represents a single eye, with error bars showing the 95% confidence intervals on *C*_*r*_ arising from the regression analysis. For each condition, the predicted log-normal distribution is shown, with the thick central white band showing the geometric mean and the thinner white bands showing two geometric standard deviations from the mean. The shaded central region indicates the 95% confidence interval on the mean. **(b)** Paired facility plot: each data point represents one pair of eyes, with *C*_*r*_ for the treated T siRNA eye on the Y-axis and the *C*_*r*_ for contralateral control NT-siRNA eye on the X-axis. The red line shows the average difference between contralateral eyes, with its confidence interval in grey, whilst the blue line represents the case of identical facility between contralateral eyes, corresponding to no effect due to T siRNA. All data points are above the blue unity line, indicating that the facility was higher in the treated eyes compared to the controls; n = 7, p = 0.006. Inner blue ellipses show the 95% confidence intervals on *C*_*r*_ arising from the regression analysis, whilst the green outer ellipses show additional uncertainty due variability between contralateral eyes, estimated from 10 pairs of C57BL/6J eyes perfused only with glucose supplemented PBS^36^.

In order to evaluate whether down-regulation of TJs increases outflow facility, studies were performed in mouse eyes since the conventional outflow pathway of mice resembles that of human morphologically, physiologically and pharmacologically^33^,^34^,^35^. We targeted ZO-1 and tricellulin based on the IHC data obtained in Fig. 3a. Seven wild type C57BL/6J mice were intracamerally injected with a combination of 1 μg ZO-1 siRNA and 1 μg of tricellulin siRNA, and contralateral eyes were injected with 2 μg of NT siRNA. 48 h post-injection, all animals were sacrificed and enucleated eyes were perfused in pairs using the recently developed *iPerfusion*^36^ system to measure outflow facility, calculated from the flow measured over multiple pressure steps (Supplementary Fig. S9). Outflow facility in the siRNA treated eyes was increased compared to eyes receiving NT siRNA (Fig. 7a). Figure 7b shows the paired facility data where the facility of the treated eye is plotted against that of the contralateral control eye. In all cases, the facility of the treated eye was elevated compared to control (n = 7 pairs), exhibiting an average facility increase of 113% (confidence interval [35, 234]%, p = 0.0064, facility values for each pair of eyes are provided in Supplementary Table S2). These data demonstrate that down-regulation of TJ components within the conventional outflow pathway significantly increases conventional outflow facility in mouse eyes *ex vivo*. To investigate the long-term effect of TJ down-regulation, we perfused eyes from animals 8 weeks post-injection and we observed no difference in outflow facility between treated and control eyes (average facility increase of 9.2%, confidence interval [−14.22, 32.62]%, p > 0.05, n = 4 pairs, facility values for each pair of eyes are provided in Supplementary Table S2). This observation indicates that a single injection of siRNAs enables transient and reversible modulation of outflow facility in the anterior chamber of murine eyes.

## Discussion

AH exiting the anterior chamber via the conventional outflow pathway passes through the tissues of the TM and into the SC lumen by crossing its endothelial barrier. Conceptually, loosening the TJs that bind endothelial cells could render the barrier more permeable resulting in reduced outflow resistance. However, this targeted approach has not been previously assessed in regard to AH outflow. The current study focused on identifying TJ components present in human, murine and non-human primate outflow tissues that might serve as plausible targets for siRNA-mediated down-regulation. A number of such targets were identified in primary cultures of human SCEC, disruption of which has previously been associated with altering endothelial cell permeability in other cell systems^37^,^38^,^39^.

The TJ profile found in human SCEC identifies claudin-11 and tricellulin as SC specific TJ related proteins not present in TM cells, while ZO-1 has been found to be present in both SCEC and TM cells as previously reported^29^,^40^. Previous studies have also reported the identification of specific protein markers that are either exclusively expressed in the inner wall endothelium of SC, or differ appreciably in their expression from TM cells^14^,^28^,^41^,^42^. Owing to the high level of claudin-11 expression found in SCEC as compared to TM cells, this claudin-based TJ may also be used as a specific marker for identifying SC cells.

The association of both claudin-11 and tricellulin with SCEC is of significance because both TJs have been associated with maintenance of barrier function. Several studies on paracellular tightness have demonstrated that claudin-11 modulates paracellular cation permeability^39^,^43^,^44^ and its knockdown increases TEER in human corpus cavernosum endothelial cells^45^. On the other hand, transcript knockdown studies have shown that the inhibition of tricellulin leads to instability of TJs^46^, whereas tricellulin overexpression is associated with reduced permeability to macromolecules^47^. Accordingly, we observed that siRNA-mediated knockdown of selected TJ decreases transendothelial resistance and increases permeability to 70 kDa FD in cultured human SC cell monolayers. In particular, these effects were more profound when a combination of siRNAs was used, suggesting a synergistic effect in increasing paracellular permeability following down-regulation of a range of TJs.

Paracellular pathways have been well established to possess defined values of electrical conductance as well as charge and size selectivity^48^. For example, the junctional complexes comprising of tight and adherens junctions between cerebral endothelial cells enable the blood-brain barrier to regulate the entry of blood-borne molecules and preserve ionic homeostasis within the brain microenvironment^49^. Our size selectivity data indicate that SC endothelial barriers have restrictive properties to regulate paracellular passage, and direct alteration of TJ complex only allows the passage of dextrans of up to 70 kDa, representing a biologically relevant size comparable to albumin (66 kDa), which does not cross the paracellular route readily in unperturbed endothelial monolayers^50^. We have shown that TJ barriers are formed and localised along the endothelial cells of the inner wall of SC *in vivo*. In conjunction with *in vitro* permeability data, TJs in the inner wall endothelium of SC are identified as possibly playing a pivotal role in contributing to paracellular movement of AH and solutes across the endothelial layer. Similarly to human SCEC, non-human primates also express ZO-1, claudin-11 and tricellulin in the inner wall of SC. However, we did not detect claudin-11 expression in the mouse outflow pathway, which suggests differential expression patterns between species.

In order to prove the efficacy of the siRNA in an *in vivo* system, ZO-1 and tricellulin siRNA were injected into the anterior chambers of mice. We show that knockdown of transcripts encoding TJs in the conventional outflow pathway increases AH outflow facility in wild type mice, and that this effect is associated with the presence of an increased number of open intercellular clefts between SCEC. It is therefore reasonable to infer that opening of intercellular clefts is responsible for the increased outflow facility measured *ex vivo*. In contrast to studies using EDTA to disrupt cellular junctions along the inner wall^31^,^32^, no eyes treated with siRNA exhibited signs of necrosis or apoptosis, and there were no platelets adhering to the inner wall. This indicates that the endothelial cell membranes remained intact and the subendothelial ECM was not exposed to the lumen of SC.

Our *in vivo* data also reinforce that the hydraulic conductivity of the inner wall endothelium of SC is maintained by the adhesive forces produced at the endothelial cell-cell junctions between TJ proteins^14^,^28^. It is therefore correct to propose that factors which change the adhesive properties of TJ proteins in the inner wall of SC may alter the existing behaviour of the outflow pathway. To illustrate this point, we have preliminary data demonstrating higher claudin-11 and ZO-1 expression in glaucomatous SCEC monolayers as compared to healthy controls (Supplementary Fig. S10a). In addition, cultured glaucomatous SCEC strains displayed higher TEER values than healthy strains (Supplementary Fig. S10b). The increase in TJ expression found in glaucomatous SCEC suggests that altered barrier function in the inner wall of SC may negatively impact on conventional outflow behaviour.

While conventional adeno-associated viruses (AAV) have been shown to be inefficient in transducing cells of the outflow tissues, self-complementary AAV have been reported to be effective in such transduction^51^,^52^. It is of note that AAV expressing inducible short hairpin RNAs (shRNA) targeting claudin-5, or a combination of claudin-5 and occludin have been used to transfect cerebral and retinal tissues, and that down-regulation of these TJ vascular endothelial cell components renders the blood-brain and inner blood-retina barriers reversibly permeable to compounds up to 1 kDa, or 5 kDa respectively^53^,^54^. Should it prove possible using this technique to periodically activate virus expressing shRNAs within SCEC using an inducible promoter, expression of such shRNA could in principle be used as a means of periodically increasing outflow facility in cases of POAG in which patients fail to achieve target IOP with conventional medications. Alternatively, episcleral delivery of siRNA, where materials can be delivered non-invasively into the outflow tissues in a retrograde fashion as an outpatient procedure^55^, might represent an attractive alternative, thus avoiding the necessity of introducing a viral vector into the anterior chamber to secure viral-mediated shRNA expression. To explore the feasibility of an episcleral delivery approach, we have successfully achieved delivery of biotin conjugated tracer molecules to the conventional outflow pathway via the episcleral route in mice. Taken together, results from this study support the concept that endothelial TJs of the inner wall of SC are an attractive target upon which to base future attempts to increase AH outflow in cases of ocular hypertension.

## Materials and Methods

### Cell Culture

Human SCEC and TM cells were isolated, cultured and characterised as previously described^56^,^57^. SCEC strains used in this study were SC65, SC68, SC73, SC76, SC77, SC82 and SC83. TM93 was used for RNA analysis, whereas TM120 and TM130 were used for protein analysis. All SCEC and TM cells were used between passages 2 and 6. SCEC were cultured in low glucose Dulbecco’s modified Eagle medium (Gibco, Life Sciences) supplemented with 10% *Performance Plus* foetal bovine serum (FBS) (Gibco, Life Sciences), 1% Pen/Strep glutamine (Gibco, Life Sciences), in a 5% CO_2_ incubator at 37 °C. TM cells underwent a differentiation step by plating at full confluency for one week in media containing 10% FBS, and changed over to media containing 1% FBS for an additional week prior to experimentation. Cultured cells were passaged with trypsin-EDTA (Gibco-BRL) to maintain exponential growth.

### Human tight junction PCR array

The human TJ RT^2^ Profiler PCR array (PAHS-143ZA, Qiagen) was used to profile the expression of 84 key genes encoding proteins that form selective barriers between epithelial and endothelial cells to regulate size selectivity, polarity, proliferation and differentiation. Total RNA was extracted from four different human SCEC strains (SC65, 68, 76 and 77) at passages 3 to 5 using RNEasy Mini Kit (Qiagen) according to manufacturer’s protocol. Genomic DNA contamination was eliminated by DNase treatment. Total RNA was reverse-transcribed into cDNA using RT^2^ First Strand Kit (Qiagen). The Threshold cycle (Ct) values of different passage numbers from each SCEC strain were determined and averaged using ABI Prism 7700 Sequence Detector. The mean normalised expression (2^−∆Ct^) of genes encoding claudin and adhesion junctional proteins was determined and analysed using the online Qiagen RT^2^ Profiler PCR Array Data Analysis software. Normalised gene expression was calculated by using the equation: 2^−∆Ct^ = 2^−[Ct(gene of interest)−Ct(Housekeeping genes)]^. Normalisation was carried out with five housekeeping genes (*ACTB, B2M, GAPDH, HPRT1 and RPLP0*) included in the PCR array. The 2^−∆∆CT^ = 2^−∆Ct treated^/2^−∆Ct control^ method was used to calculate fold changes for each gene as difference in gene expression^58^.

### Western Blot

Protein lysates were isolated from cultured cells in protein lysis buffer containing 1 M Tris pH 7.5, 1 M NaCl, 1% NP-40, 10% SDS, 1X protease inhibitor cocktail (Roche). The homogenate was centrifuged at 10,000 r.p.m. (IEC Micromax microcentrifuge, 851 rotor) at 4 °C for 20 min and the supernatant was stored at −80 °C until use. Protein concentration was determined by BCA Protein assay kit (Pierce, IL, USA) with bovine serum albumin (BSA) at 2 mg/ml as standards on 96-well plates according to the manufacturer’s protocol. 30–50 μg of total protein was loaded in each lane. Protein samples were separated by electrophoresis on 7.5–10% SDS–PAGE under reducing conditions and electro-transferred to PVDF membranes. After blocking with 5% blotting grade blocker non-fat dry milk in TBS for 1 h at room temperature, membranes were incubated overnight at 4 °C with the following Rabbit polyclonal primary antibodies: anti-oligodendrocyte specific protein antibody (1:500; Abcam); anti-ZO-1 antibody (1:250; Invitrogen), anti-tricellulin C-terminal antibody (1:125; Invitrogen), anti-occludin antibody (1:500, Invitrogen) and anti-VE-cadherin antibody (1:1000; Abcam). Blots were washed with TBS and incubated with horse radish peroxidase-conjugated polyclonal rabbit IgG secondary antibody (Abcam). The blots were developed using enhanced chemiluminescent kit (Pierce Chemical Co.) and exposed to Fuji X-ray film. Each blot was stripped with Restore Western Blot Stripping Buffer (Pierce) and probed with rabbit polyclonal to β-actin or GAPDH (Abcam) as loading controls. Protein band intensities were quantified by scanning with a HP Scanjet Professional 10000 Mobile Scanner and analysed using *Image J* (Version 1.50c). The percentage reduction in band intensity was calculated relative to the control non-targeting siRNA, which was standardised to represent 100% and normalised against β-actin.

### Immunocytochemistry

Human SCEC were grown on Lab-Tek II chamber slides and fixed in 4% paraformaldehyde (pH 7.4) for 20 min at room temperature and then washed with PBS for 15 min. Cell monolayers were blocked in PBS containing 5% normal goat serum and 0.1% Triton X-100 at room temperature for 20 min. Primary antibodies were diluted at 1:100 in blocking buffer and incubated overnight at 4 °C. Secondary antibodies diluted at 1:500 were then incubated for 2 h at room temperature in a humidity chamber. Following incubation, chamber slides were mounted with aqua-polymount (Polyscience) after nuclei-counterstaining with DAPI. Fluorescent images of SCEC monolayers were captured using a confocal microscope (Zeiss LSM 710), and processed using imaging software ZEN 2012.

### Immunohistochemistry for frozen sections

Enucleated mouse eyes were fixed in 4% paraformaldehyde (pH 7.4) overnight at 4 °C on a rotating device. Posterior segments of the eye and the lens were removed and anterior segments were then washed with PBS for 15 min and sequentially submerged in 10, 20 and 30% sucrose. Dissected anterior segments were then suspended in specimen blocks with OCT solution (Tissue-Tek) and frozen in a bath of isopropanol submerged in liquid nitrogen. Frozen anterior segments were sectioned using a cryostat (Leica CM 1900) to 12 μm thickness. Sections were collected on Polysine^®^ slides (Menzel-Glazer). To detect TJ proteins, sections were blocked for 20 min at room temperature in PBS containing 5% goat serum and 0.1% Triton-X, and incubated with the corresponding antibodies at 1:100 dilutions overnight at 4 °C in a humidity chamber. All sections were then washed three times in PBS and incubated with Cy-3 labelled anti-rabbit IgG antibody at 1:500 (Abcam) for 2 h at room temperature in a humidity chamber. Following incubation, sections were washed with PBS and mounted with aqua-polymount (Polyscience) after nuclei-counterstaining with DAPI. Anterior segments were visualised using a confocal microscope (Zeiss LSM 710).

### Immunohistochemistry for paraffin embedded sections

Paraffin sections of African green monkey (*Chlorocebus Sabeus*) anterior segments were rehydrated by immersion in the following solutions: twice for 2 min each in Histoclear solution; 100% ethanol for 1 min; 95% ethanol for 1 min; 70% ethanol for 1 min; deionised water for 1 min; washing twice for 5 min in PBS. For antigen retrieval, paraffin sections were heated to 95 °C for 10 min in citrate buffer (Sodium citrate, pH 6). Paraffin sections were then blocked and stained as described above.

### siRNAs

All *in vivo* predesigned siRNAs used in this study were synthesised by Ambion and reconstituted as per manufacturer’s protocol. siRNA identification numbers are as follows: human claudin-11 siRNA (ID number: s9925), human ZO-1 siRNA (ID number: s14156), human MARVELD2 siRNA (ID number: s45794), mouse ZO-1 siRNA (ID number: s75175), mouse MARVELD2 siRNA (ID number: ADCSU2H). Silencer Negative control siRNA (Ambion) was used as a non-targeting control in knockdown studies.

### Cell viability assay

SCEC were grown to confluency on a 96-well plate. Cells were transfected with siRNA in quadruplicate using Lipofectamine RNAiMax reagent as outlined by the manufacturer (Life Technologies) at both 1 pmol/well (40 nM) and 5 pmol/well (200 nM). Cells were left for 48 hours, apart from a media change after 24 hours. CellTitre 96 AQueous One Solution Reagent (Promega) was thawed and mixed with culture medium at a 1:5 dilution. Cells were incubated with this mixture for a period of 2 hours, before transferring the media to a fresh 96-well plate. Absorbance of each well was recorded at 450 nm on a spectrophotometer (Multiskan FC, THermo Scientific). After blanking against wells with reagent and no cells, each treatment group was presented relative to a negative control containing no siRNA. A positive control was achieved by incorporating 1% SDS into the media-reagent mixture. A one-way ANOVA with a Tukey’s post-test was performed on the data set.

### Measurement of SCEC monolayer transendothelial electrical resistance (TEER)

TEER was used as a measure of TJ integrity by the human SCEC monolayers as previously descrived^54^. In brief, human SCEC (1 × 10^4^ cells per well) were grown to confluency on Costar HTS Transwell-polyester membrane inserts with a pore size of 0.4 μm. The volume of the apical side (inside of the membrane inserts) was 0.1 ml and that of the basal side (outside of the membrane inserts) was 0.6 ml. Confluent cells were then transfected in triplicates with 40 nM of claudin-11, ZO-1 and tricellulin siRNAs, or in combination, using Lipofectamine RNAiMax reagent as outlined by the manufacturer (Life Technologies). Non-targeting siRNA was used as a control. 48 h post-transfection, TEER values were determined using an EVOM resistance meter with Endohm Chamber (World Precision Instruments) and a Millicell-Electrical Resistance System. For measurement of TEER, both the apical and basolateral sides of the endothelial cells were bathed in fresh growth medium at 37 °C, and a current was passed across the monolayer with changes in electrical resistance, which was reported as Ω.cm^2^ after correcting for the surface area of the membrane (1.12 cm). Electrical resistance was measured in triplicate wells, and the inherent resistance of a blank transwell was subtracted from the values obtained for the endothelial cells.

### Cell permeability assay using FITC-dextran

Human SCEC were prepared and treated using the same method for TEER measurement as described above. Transwell permeability assays were carried out as previously described^54^. In brief, 4 kDa, 70 kDa and 150 kDa fluorescein isothiocyanate (FITC)-conjugated dextran (FD) (Sigma) was applied at 1 mg/ml to the basal compartment of the transwells. Sampling aliquots of 0.1 ml were collected every 15 min for a total of 120 min from the apical side for fluorescence measurements and the same volume of culturing media was added to replace the medium removed. FITC fluorescence was determined using a spectrofluorometer (Optima Scientific) at an excitation wavelength of 485 nm and an emission wavelength of 520 nm. Relative fluorescence units (RFU) were converted to values of nanograms per millilitre using FITC-dextran standard curves, and were corrected for background fluorescence and serial dilutions over the course of the experiment. The apparent permeability co-efficient (*P*_*app*,_ cm/s) for each treatment was calculated using the following equation:


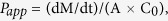


where dM/dt (μg/s) is the rate of appearance of FD on the apical side from 0 min to 120 min after application of FD. C_0_ (μg/ml) is the initial FD concentration on the basal side, and A (cm^2^) is the effective surface area of the insert. dM/dt is the slope calculated by plotting the cumulative amount of (M) versus time.

### Animal Husbandry

The use of animals and injections carried out in this study were in accordance with the European Communities Regulations 2002 and 2005 and the Association for Research in Vision and Ophthalmology statement for the use of Animals in Ophthalmic and Vision Research, and was approved by the institutional Ethics Committee. In this case, all procedures carried out at Imperial College London and Trinity College Dublin were approved by the UK Home Office and by the Health Products Regulatory Authority of the Irish Medicines Board (project authorisation AE19136/P017) respectively. Male C57BL/6J mice (Charles River Laboratories, UK) of age 10 to 12 weeks were used. *Ex vivo* perfusions and intracameral injections were done under the UK Home Office Project at Imperial College London. Animals were brought into the animal facility one week prior injections for an acclimatisation period. Mice were housed in individually ventilated cages with 5 mice per cage. They were provided with food and water *ad libitum* and were under 12 h light/dark cycles (7 am to 7 pm) at 21 °C.

### Intracameral injection

Adult C57BL/6J mice of 10 to 12 weeks of age were anaesthetised by intra-peritoneal injection of medetomidine hydrochloride (Domitor) and ketamine (0.66 and 66.6 mg/kg body weight, respectively). Pupils were dilated with 2.5% tropicamide and 2.5% phenylephrine eye drops. Glass micro-capillaries (outer diameter = 1 mm, inner diameter = 0.58 mm; World Precision Instruments) were pulled using a micropipette puller (Narishige PB-7). Under microscopic control, a pulled blunt-ended micro-glass needle (tip diameter ~100 μm) was first used to puncture the cornea to withdraw AH. Immediately after puncture, a pulled blunt-ended micro-glass needle attached to a 10 μl syringe (Hamilton, Bonaduz) was inserted through the puncture, and 1.5 μl of PBS containing 1 μg of ZO-1 siRNA and 1 μg of tricellulin siRNA was administered into the anterior chamber to give a final concentration of 16.84 μM. Contralateral eyes received an identical injection of 1.5 μl containing the same concentration of NT siRNA. Following surgery, a reversing agent (1.5 mg/kg body weight, atipamezole hydrochloride) was delivered by intra-peritoneal injection. Fusidic gel was applied topically to the eye as antibiotic and Vidisic gel was also applied topically as a moisturiser. Furthermore, 5 mg/kg enrofloxacin antimicrobial (Baytril; Bayer Healthcare) was injected subcutaneously.

### Apoptosis markers staining

IHC was performed on perfused eyes 48 hours post injection with either targeting or non-targeting siRNA. Approximately 30–40 12 μm sections were each stained by TUNEL and complemented by cleaved caspase-3 staining as markers of apoptosis for representatives of targeting and non-targeting siRNA. Eyes were fixed and prepared for cryosectioning as described before. TUNEL staining was performed as per manufacturers protocol (*in situ* Cell Death Detection Kit, POD, Roche), for positive controls, slides treated with Dnase-1 for 10 minutes at room temperature after permeabilisation and prior to antibody labelling. Cleaved caspase-3 staining was performed as described before using cleaved caspase 3 marker antibody (#9661, Cell Signalling Technology), positive controls were obtained by perfusing wild type eyes *ex vivo* for 24 hours at 35 °C with 200 ng/ml of mouse IL-1B and 100 ng/ml of human TNF-**α** in DMEM to induce apoptosis.

### Transmission electron microscopy (TEM)

All eyes were immersion fixed in Karnovsky’s solution initially and post-fixed in Ito’s solution. The eyes were embedded in Epon and semi-thin sagittal sections were cut through the whole globe. Ultrathin sections of SC and TM were cut sagitally from one side of the eye first, and then another ultrathin section approximately 1mm deeper was cut. If possible, this section was taken from the other side of the eye. In the small mouse eye, this process could be repeated four times. In this way, different parts of the circumference of the eye were evaluated. Different staining methods were investigated to visualise cell membranes, and the best results were obtained using UAR-EMS (Science Services, Munich, Germany). In ultrathin sections of the entire anterior posterior length of the inner wall from all four regions of treated eyes and their controls, we investigated whether there was any ballooning of the inner wall endothelium, necrosis or apoptosis of endothelial cells or adherence of platelets to the inner wall endothelium. Intercellular gaps were counted at magnifications of 80.000x by two independent observers (ELD and CFK).

### Outflow facility measurements

Mouse eyes were perfused *ex vivo* to measure outflow facility using the *iPerfusion* system^36^. Mice were culled by cervical dislocation and the eyes were enucleated within 10 min post mortem and stored in PBS at room temperature to await perfusion (~20 min). Both eyes were perfused simultaneously using two independent perfusion systems as described previously^36^. Briefly, each eye was affixed to a support using a small amount of cyanoacrylate glue and submerged in a PBS bath regulated at 35 °C. The eye was cannulated via the anterior chamber with a 33-gauge bevelled needle (NanoFil, #NF33BV-2, World Precision Instruments) under a stereomicroscope using a micromanipulator. The *iPerfusion* system comprises an automated pressure reservoir, a thermal flow sensor (SLG64-0075, Sensirion) and a wet-wet pressure transducer (PX409, Omegadyne) in order to apply a desired pressure, measure flow rate out of the system and measure the intraocular pressure respectively. The perfusate was DBG (PBS including divalent cations and 5.5 mM glucose), and was filtered through a 0.22 μm filter (VWR international) prior to use.

Following cannulation, eyes were perfused for 30 min at ~8 mmHg to allow the eye to acclimatise to the environment. Subsequently, nine discrete pressure steps were applied from 4.5 to 21 mmHg, while flow and pressure were recorded. Stability was defined programmatically, and data were averaged over 4 min at steady state. A non-linear model was fit to flow-pressure data to account for the pressure dependence of outflow facility in mouse eyes. This model was of the form *Q* = *C*_*r*_ *P (P/P*_*r*_)^*β*^. where *Q* and *P* and are the flow rate and pressure respectively, and *C*_*r*_ is the outflow facility at reference pressure *P*_*r,*_ which is selected to be 8 mmHg (the approximate physiological pressure drop across the outflow pathway). The power law exponent *β* quantifies the non-linearity in the *Q-P* response and thus the pressure dependence of outflow facility. The data analysis methodology described previously^36^ was applied in order to analyse the treatment effect, whilst accounting for measurement uncertainties and statistical significance was evaluated using the paired weighted *t*-test described therein.

### Statistical analysis

For real-time PCR, TEER and paracellular permeability measurements, Student’s *t*-tests and ANOVA with Bonferroni post-test were carried out using GraphPad Prism 5.0. For *ex vivo* perfusions, a paired weighted *t*-test was performed using MATLAB as described^36^. For open clefts quantification, an unpaired *t*-test was performed. Statistical significance was indicated by *p* ≤ 0.05.

## Additional Information

**How to cite this article**: Tam, L. C. S. *et al*. Enhancement of Outflow Facility in the Murine Eye by Targeting Selected Tight-Junctions of Schlemm’s Canal Endothelia. *Sci. Rep.* **7**, 40717; doi: 10.1038/srep40717 (2017).

**Publisher's note:** Springer Nature remains neutral with regard to jurisdictional claims in published maps and institutional affiliations.

## Supplementary Material

Supplementary Data

## Figures and Tables

**Figure 1:**
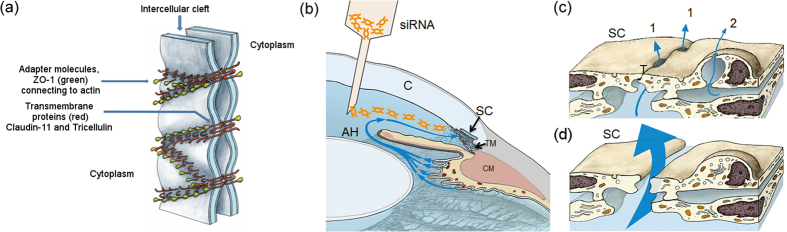
Schematic illustration of the therapeutic strategy addressed in this study. **(a)** Schematic representation of adapter molecules and transmembrane proteins connecting neighbouring SCEC. **(b)** Intracameral delivery enables siRNAs to be transported towards the conventional outflow pathway by following the natural flow dynamics of aqueous humour in the anterior chamber. AH = aqueous humour; C = cornea; CM = ciliary muscle; SC = Schlemm’s canal; TM = trabecular meshwork. **(c)** AH crosses the inner wall endothelium of SC via (1) the intercellular pathway through gaps in tight junctions (T) and, or via (2) the intracellular pathway through a giant vacuole with a pore. **(d)** siRNAs taken up by endothelial cells of the inner wall of SC elicit knockdown of tight junction proteins, resulting in the opening of intercellular clefts with concomitant increase in aqueous outflow facility.

**Table 1:** Quantification of total and open intercellular clefts along the inner wall in treated eyes that were immersion fixed immediately after death.

Sample	Region 1		Region 2		Region 3		Region 4		% open
	N total	N open	N total	N open	N total	N open	N total	N open	
C4 NEP T	15	4	17	3	21	5	24	9	27%
C4 REP T	18	15	14	3	22	17	10	5	63%
C4 LEP T	9	5	26	1	18	9	12	3	28%
C5 LEP T	26	10	15	3	24	4	20	4	25%
C6 LEP T	25	11	31	11	15	4	18	2	31%
C6 NEP T	25	2	29	9	16	4	30	8	23%
								Average	33%
								SD	25%

**Table 2:** Quantification of total and open intercellular clefts along the inner wall in control eyes that were immersion fixed immediately after death.

Sample	Region 1		Region 2		Region 3		Region 4		% open
	N total	N open	N total	N open	N total	N open	N total	N open	
C4 NEP NT	8	0	22	0	17	2	28	0	3%
C4 REP NT	17	1	20	0	13	0	18	0	1%
C5 LEP NT	21	0	26	0	14	0	19	0	0%
C6 LEP NT	11	1	30	0	26	0	9	0	1%
C6 NEP NT	20	1	17	0	29	0	31	1	2%
								Average	2%
								SD	1%
