# Supplementary material for: Enhancement of Outflow Facility in the Murine Eye by Targeting Selected Tight-Junctions of Schlemm’s Canal Endothelia
Source: Sci Rep. 2017 Jan 16;7:40717. doi: 10.1038/srep40717 (PMC5238500; doi:10.1038/srep40717)
Supplement: Supplementary Data [file srep40717-s1.pdf]

ENHANCEMENT OF OUTFLOW FACILITY IN THE MURINE EYE BY TARGETING SELECTED TIGHT-JUNCTIONS OF SCHLEMM'S CANAL ENDOTHELIA

Lawrence C.S. Tam[1*+], Ester Reina-Torres[1,2+], Joseph M. Sherwood[2], Paul S. Cassidy[1], Darragh E. Crosbie[1], Elke Lütjen-Drecoll [3], Cassandra Flügel-Koch[3], Kristin Perkumas[4], Marian M. Humphries[1], Anna-Sophia Kiang[1], Jeffrey O'Callaghan[1], John J. Callanan[5], A. Thomas Read[6], C. Ross Ethier[7], Colm O'Brien[8], Matthew Lawrence[9], Matthew Campbell[1], W. Daniel Stamer[4*], Darryl R. Overby[2], Pete Humphries[1*]

[1]Ocular Genetics Unit, Institute of Genetics, Trinity College, University of Dublin, Dublin 2, Ireland.

[2]Department of Bioengineering, Imperial College London, London, UK.

[3]Department of Anatomy, University of Erlangen-Nürnberg, Erlangen, Germany. [4]Department of Ophthalmology, Duke University, Durham, NC, USA.

[5]Ross University School of Veterinary Medicine, P. O. Box 334, Basseterre, St. Kitts, West Indies.

[6]Department of Ophthalmology and Vision Sciences, University of Toronto, Canada. [7]Coulter Department of Biomedical Engineering, Georgia Institute of Technology and Emory University, Atlanta, USA.

[8]Ophthalmology, Mater Hospital, UCD School of Medicine, Dublin, Ireland.

[9]RxGen, Hamden, CT, USA.

*Correspondence should be addressed to L.C.S.T (lawrenct@tcd.ie), P.H. (pete.humphries@tcd.ie) or W-D.S (william.stamer@duke.edu)

[+]These authors contributed equally to this work.

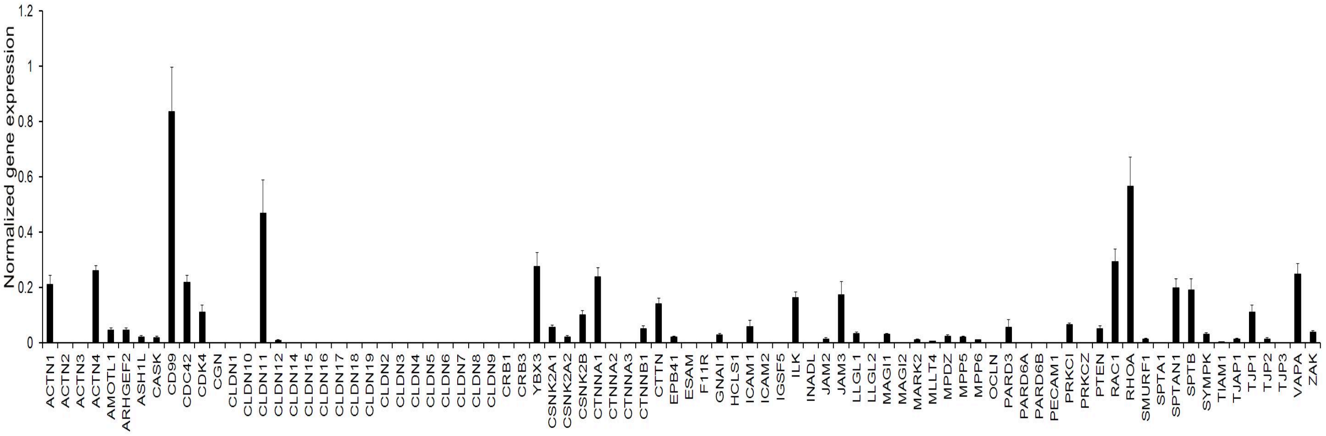

Supplementary Figure S1: A complete normalised gene expression pattern of human TJs in cultured human SCEC. SC65, 68, 76 and 77 SCEC strains were used for this study. Data are mean ± s.e.m.

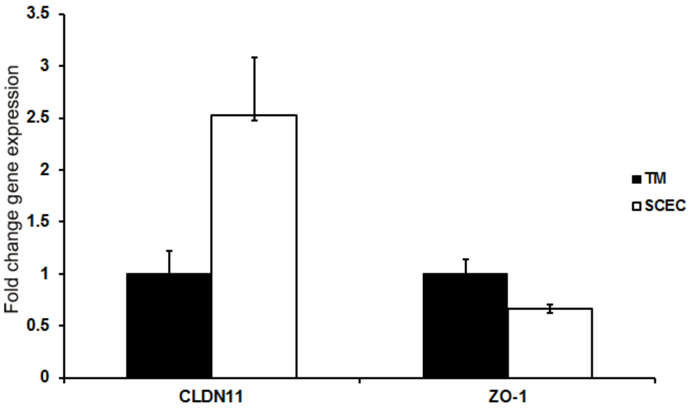

Supplementary Figure S2: Comparison of claudin-11 and ZO-1 gene expression between cultured human TM and SCEC. Fold change in gene expression was determined by the $2^{-\Delta\Delta Ct}$ method. Data represent mean fold change of SC77 and TM93 cell strains at two-passage numbers ± s.e.m (n = 2).

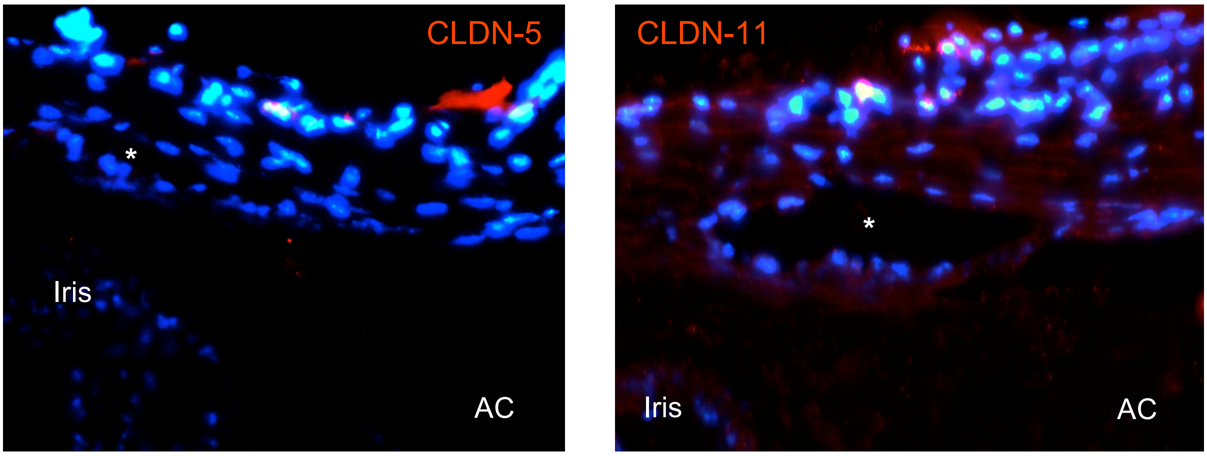

Supplementary Figure S3: Immunostaining of claudin-5 and claudin-11 in frozen sections of mouse anterior segments. claudin-5 and claudin-11 = Cy3 (red); DAPI = blue; * = Schlemm's canal lumen.

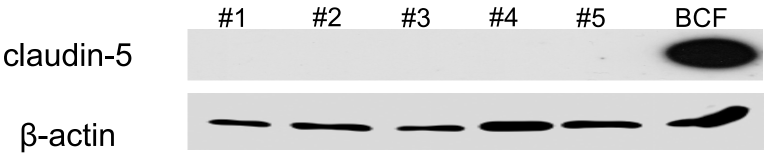

Supplementary Figure S4: Western blot for claudin-5 in Schlemm's canal endothelial cells dissected from five different non-human primates. BCF = mouse brain capillary fraction as positive control.

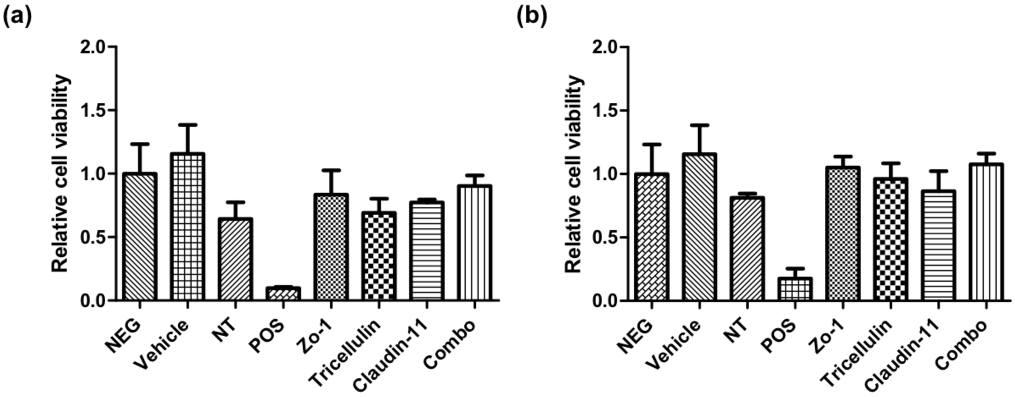

Supplementary Figure S5: Cell viability essay on cultured SCEC treated with **(a)** 40 nM siRNA or **(b)** 200 nM siRNA. No significant difference was found between the negative control and any of the siRNA treated samples (n=4; one-way ANOVA with a Tukey's post-test). NEG = negative control; Vehicle = transfection reagents alone; NT = non-targeting siRNA; POS = positive control; Combo = combination of ZO-1, claudin-11 and tricellulin.

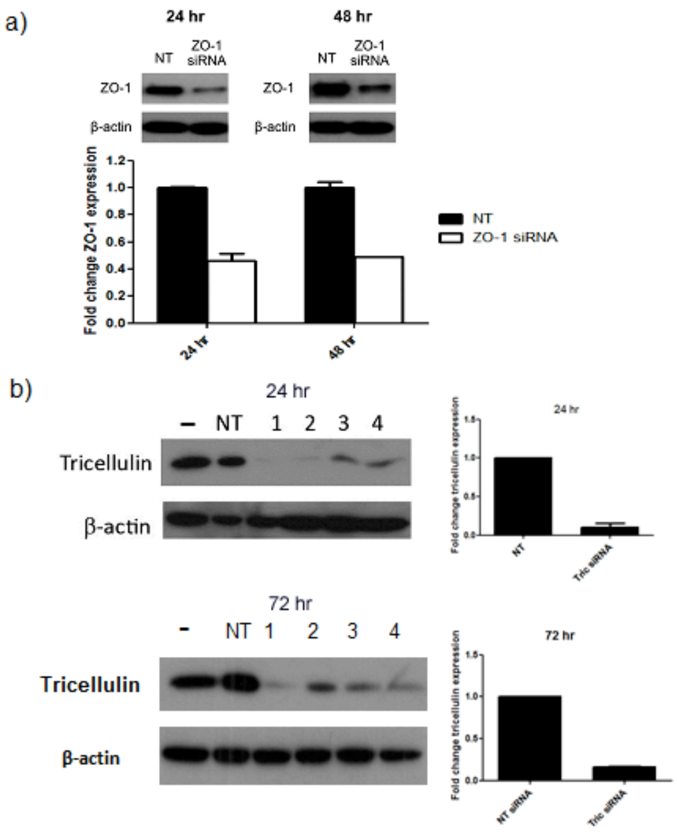

Supplementary Figure S6: a) Western blot analysis illustrating knockdown of ZO-1 in mouse brain endothelial cells (bEND3) 24 and 48 hr post-transfection. Data show average ± s.e.m. (n=2). **b)** Western blot analysis illustrating knockdown of tricellulin in HEK293 cells transfected with mouse tricellulin cDNA 24 and 72 hr post-siRNA transfection (n=4). NT: non-targeting siRNA.

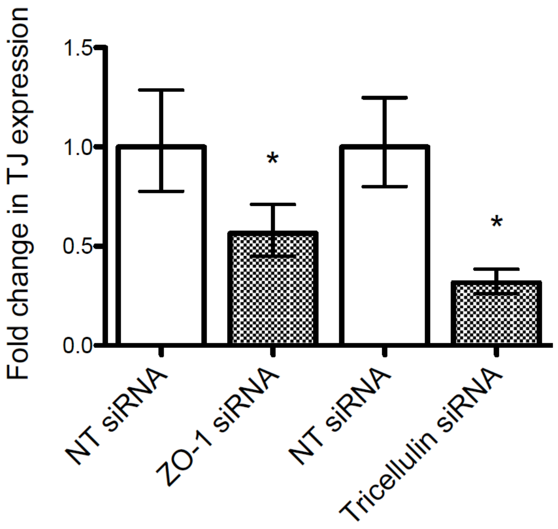

Supplementary Figure S7: Quantitative RT-PCR analysis illustrating knockdown of ZO-1 and tricellulin transcript levels at 12 hrs post siRNA delivery in the mouse retina. n = 3, *P≤0.05. Data is mean fold change with max and min from ΔΔCt standard deviations.

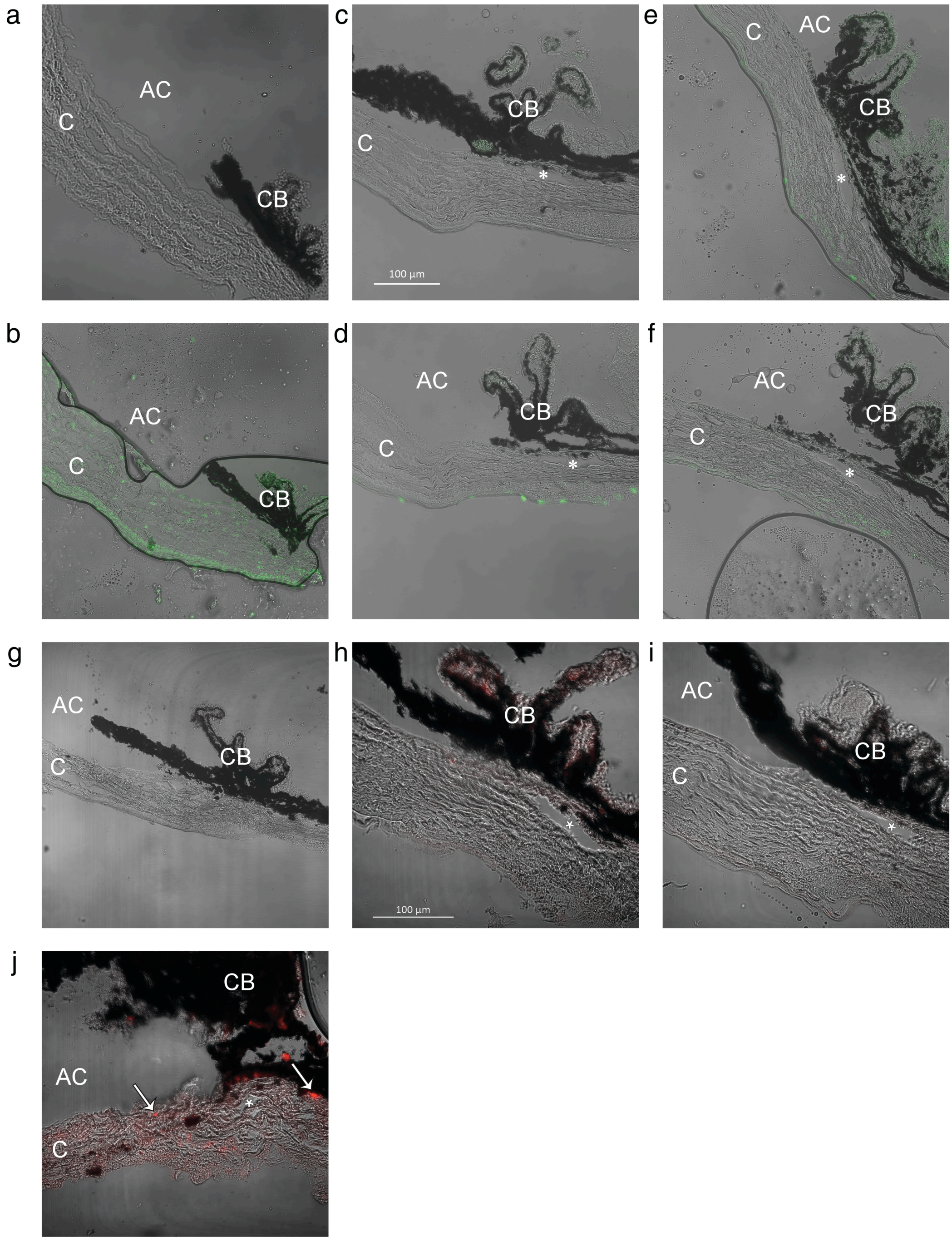

Supplementary Figure S8. (**a-f**) IHC based TUNEL staining. (**a**) Negative control. (**b**) DNAase1 treated positive control. (**c-d**) Representative non-targeting images showing minor fluorescence in the corneal epithelium and ciliary body. (**e-f**) Representative targeting images showing similar staining patterns to that of non-targeting controls. (**g-i**) Cleaved caspase-3 staining. (**g**) Negative control. (**h**) Non-targeting staining shows little to no signal in outflow tissues, and minor signal in the ciliary. (**i**) Similar staining was observed in targeting sections as a whole. (**j**) Caspase-3 positive control. Representative scale bars denote 100 µm. Arrows = high incidence of apoptosis; Asterisk = SC lumen; C = cornea; AC = anterior chamber; CB = ciliary body.

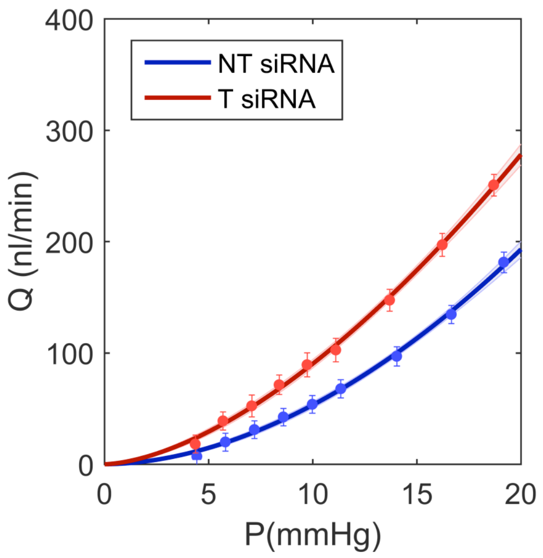

Supplementary Figure S9: Representative flow (Q) *vs.* pressure (P) plot for a pair of eyes. Each data point shows the average of 4 min of stable flow at each pressure step, and error bars represent 95% confidence intervals. A power-law model (see Methods) is fit to the data and the 95% confidence bounds of the fit are represented by the shaded areas.

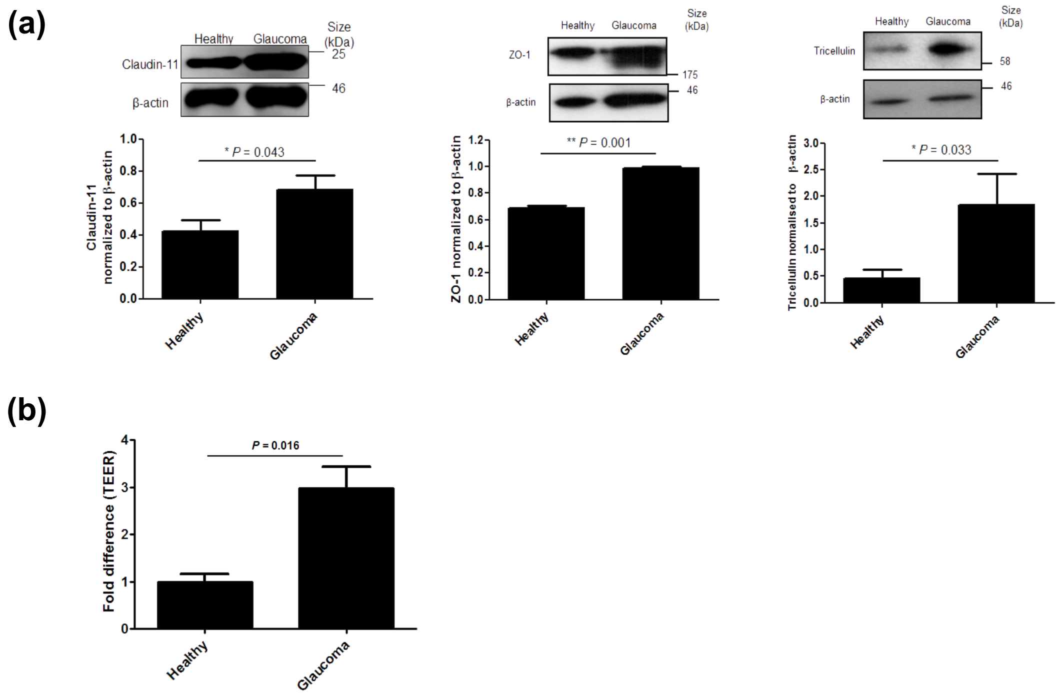

Supplementary Figure S10. (a) Western blots comparing Claudin-11, ZO-1 and Tricellulin protein expression between cultured healthy and glaucomatous SCEC monolayers. Histograms depict relative densitometric changes in protein expression from four independent experiments. **(b)** TEER values were measured in cultured glaucomatous SCEC strain (SC57g) and healthy SCEC strain (SC68) after one week of confluency was reached. Data represented as mean ± s.e.m. (unpaired Student's *t* test).

Time post-transfection (hr)	NT siRNA (Ω.cm ± s.e.m.) n=6	CLDN11 siRNA (Ω.cm ± s.e.m.) n=6	ZO-1 siRNA (Ω.cm ± s.e.m.) n=6	CLDN11 + ZO-1 siRNA (Ω.cm ± s.e.m.) n=6
0	10.64 ± 0.33	11.48 ± 0.28	10.64 ± 0.33	10.36 ± 0.55
24	14.56 ± 0.00	12.60 ± 0.28	10.64 ± 1.00	12.88 ±0.33
48	15.96 ± 0.55	12.60 ± 0.28	11.76 ± 1.00	7.84 ± 0.00
72	15.12 ± 0.66	12.88 ± 0.00	10.08 ± 1.00	4.20 ± 0.55

Time post-transfection (hr)	NT siRNA (Ω.cm ± s.e.m.) n=4	Tricellulin siRNA (Ω.cm ± s.e.m.) n=4
0	13.22 ± 0.38	13.66 ± 0.33
24	13.66 ± 0.57	11.87 ± 1.09
48	15.12 ± 0.30	10.98 ± 0.28
72	15.57 ±0.48	11.42 ± 0.57

Time post-transfection (hr)	NT siRNA (Ω.cm ± s.e.m.) n=6	CLDN11+ZO-1+Tric siRNA (Ω.cm ± s.e.m.) n=6
0	16.32 ± 1.42	15.26 ± 0.79
24	15.75 ± 1.74	7.77 ± 1.44
48	14.88 ± 1.49	5.88 ± 0.91
72	16.00 ± 1.49	10.50 ± 1.54

Supplementary Table S1: Raw values from TEER measurements following treatment of SCEC monolayers with claudin-11, ZO-1, tricellulin, claudin-11 + ZO-1 siRNA or a combination of the three siRNAs. Data are Ω x cm^2 ± s.e.m.

	NT siRNA		T siRNA	
Mouse	Cr (nl/min/mmHg)	$^{x}/$ ME95	Cr (nl/min/mmHg)	$^{x}/$ ME95
1	4.5	1.23	12.9	1.03
2	3.5	1.08	7.6	1.12
3	6.3	1.04	9.2	1.09
4	6.7	1.12	11.9	1.12
5	4.4	1.08	7.8	1.06
6	4.4	1.18	5.6	1.08
7	3.3	1.08	18.1	1.06

Supplementary Table S2: Facility values (Cr) for each pair of eyes together with the margin of error at 95% confidence level (ME95) measured 48 hr post-injection.

	**NT siRNA**		**T siRNA**	
**Mouse**	**Cr (nl/min/mmHg)**	**$^{x}/$ ME95**	**Cr (nl/min/mmHg)**	**$^{x}/$ ME95**
1	10.2	1.18	10.8	1.11
2	13.0	1.02	11.7	1.06
3	12.6	1.08	14.8	1.04
4	13.7	1.04	16.9	1.11

Supplementary Table S3: Facility values (Cr) for each pair of eyes together with the margin of error at 95% confidence level (ME95) measured 8 weeks post-injection.
